# Prescription of pharmacotherapy and blood pressure control among hypertensive outpatients in two semi-urban hospitals in Cameroon: a cross-sectional study

**DOI:** 10.11604/pamj.2020.37.122.21156

**Published:** 2020-10-05

**Authors:** Anastase Dzudie, Messaline Fodom Fomo, Denis Georges Teuwafeu, Clovis Nkoke, Azabji Kenfack, Divine Tim Bonghaseh, Domin Ekaney, Amy Tantchou, Cabral Tantchou, Laurent Serges Etoundi Ngoa, Andre Pascal Kengne, Simeon Pierre Choukem

**Affiliations:** 1 Department of Physiology, Faculty of Medicine and Biomedical Sciences, University of Yaoundé 1, Yaoundé, Cameroon,; 2 Clinical Research Education Networking and Consultancy (CRENC), Douala, Cameroon,; 3 Department of Internal Medicine and Subspecialties, Douala General Hospital, Douala, Cameroon,; 4 Department of Internal Medicine, Buea Regional Hospital, Buea, Cameroon,; 5 Health and Human Development (2HD) Research Network, Douala, Cameroon,; 6 Baptist Hospital Mutengene, Mutengene, Cameroon,; 7 Sub-divisional Hospital Batoke, Limbe, Cameroon,; 8 Mariner Finance, Maryland, USA,; 9 Shisong Cardiac Center, Kumbo, North West Region, Cameroon,; 10 Non-communicable Disease Research Unit, South African Medical Research Council, Cape Town, South Africa,; 11 Faculty of Medicine and Pharmaceutical Sciences, University of Dschang, Dschang, Cameroon

**Keywords:** Hypertension, antihypertensive drugs, blood pressure, Cameroon

## Abstract

**Introduction:**

several international guidelines are available on drug treatment for hypertension, but the control of hypertension remains very poor in sub-Saharan Africa (SSA). We investigated the commonly prescribed antihypertensive drugs and their association with blood pressure (BP) control in adult Cameroonians.

**Methods:**

we consecutively recruited hypertensive outpatients attending the Buea and Limbe Regional Hospitals (southwest region of Cameroon). Controlled BP was defined as BP < 140/90mmHg in hypertensive patients aged 60 years or younger, diabetics or patients with chronic kidney disease or a BP < 150/90mmHg in non-diabetic hypertensive patients older than 60 years of age (JNC8).

**Results:**

of the 408 participants included (mean age 61.1 years), 67% were female. The median duration of hypertension was 6 years and the median duration of the current treatment was 22 weeks. Commonly prescribed antihypertensives were calcium channel blockers (CCB, 35.1%), thiazide/thiazide-like diuretics (TD/TLD, 26.1%) and angiotensin-converting enzyme inhibitors (ACEI, 19.5%). The median monthly cost of antihypertensive was 10279.6 CFA (approximately equal to US$ 172). Seventy percent (70%) of participants were receiving at least 2 drugs, with ACEI+TD/TLD, CCB+TD/TLD, and ACEI+CCB+TD/TLD being the most frequent combination. The rate of BP control was 52% overall, and 60% in participants on monotherapy.

**Conclusion:**

CCBs were the most prescribed single antihypertensive drugs in this setting while ACEI+TD/TLD was the most common combination. About half of patients were at target BP control levels Improving availability and affordability of these medications may improve hypertension management and control.

## Introduction

Hypertension is a major global public health problem [1] and the leading contributor to cardiovascular diseases and deaths worldwide [2, 3]. In 2010, the estimated global population with hypertension was 1.39 billion people, representing 31% of all adults [4]. It is projected that this population will increase by about 60% to a total of 1.56 billion by 2025 [5]. According to the World Health Organisation (WHO), sub-Saharan Africa (SSA) has the highest and fast-growing prevalence of hypertension [6, 7]. Over a ten year period (between 1994 and 2003), the prevalence of hypertension increased by two to five folds amongst the urban and rural populations in Cameroon and in 2015, the prevalence of hypertension was reported at 29.7% [8]. The primary goal of antihypertensive drugs prescription is to prevent the complication of elevated blood pressure (BP) and studies have shown that antihypertensive treatment can achieve 35-40% reduction in stroke, 20-25% reduction in myocardial infarction (MI) and more than 50% reduction of heart failure [9]. However, more than two-thirds of hypertensive people cannot be controlled by one drug and will require two or more drugs selected from different classes to achieve and maintain the desired BP [10, 11].

Worldwide, treatment strategies have changed and gradually moved from monotherapy to low dose combination therapy [1]. Recent guidelines recommend both CCB and ACEI or angiotensin receptor blockers (ARB) in addition to diuretics as the first-line drugs in the management of hypertension [12, 13]. Despite the existence of several guidelines for the management of hypertension [14-18], more than half of hypertensive patients do not achieve optimum BP [19, 20]. Despite this rising burden, there is no consensus on the management of hypertensive disorders across SSA countries. Treatment choices are usually adapted from guidelines from high-income countries. Thus, the prescription of antihypertensive drugs and their effectiveness vary across settings. Assessing current treatment strategies is an important step towards improving hypertension control. This study sought to determine the commonly prescribed antihypertensive drugs either in single or combination therapy and evaluate the effects on BP control in a semi-urban setting in Cameroon.

## Methods

**Study design, setting, and sampling:** we conducted a hospital-based cross-sectional study, with data collected over a period of four months (January-April 2018) at two secondary referral hospitals of the Southwest Region (SWR) of Cameroon (Buea and Limbe Regional Hospitals). The Buea Regional Hospital (BRH) has a catchment population of over 200,000 inhabitants [21], Limbe Regional Hospital (LRH) has a catchment population of over 118,210 inhabitants as of 2015 [22]. The minimum sample size (321 participants) was calculated using the formula for the prevalence study by Cochran´s [23]. We consecutively recruited all consenting hypertensive patients aged 21 years and above with a documented diagnosis of hypertension and on antihypertensive drugs for at least 15 days consulting as outpatients in these two hospitals. Participants who were pregnant or didn´t consent were excluded.

**Data collection:** an adapted questionnaire from the WHO STEPs instrument for non-communicable diseases (NCDs) risk factors assessment was used [24]. Information on socio-demographic status (age, gender, marital status, level of education and occupation), participants´ clinical history (history of dyslipidemia, diabetes, stroke, heart failure, chronic kidney disease (CKD) and ischemic heart disease (IHD), smoking, alcohol and physical activity) and physical measurements (weight, height, and BP using WHO standard operating procedures) were obtained. Information about the duration of hypertension, duration of current treatment, BP at the start of current treatment, lists of BP-lowering medications were obtained from participants´ medical records. Blood pressure measurement was done with an automated device (OMRON MIT5 Connect) with the participants in a sitting position after at least 15min rest. Three measurements were taken on the right arm 2-3min apart and the average of the second and third readings used for data analysis. Weight (to the nearest kilogram) was measured using a weighing scale while height (to the nearest 0.5cm) was measured with a wooden stadiometer. Body mass index (BMI) in kg/m^2^ was calculated as weight (kg)/[height(m)]^2^.

**Definitions:** hypertension diagnosis was retained when a participant reported having been on BP-lowering medications over the last 15 consecutive days or an elevated BP value with systolic blood pressure (SBP) ≥140mmHg and/or diastolic blood pressure (DBP) ≥90mmHg obtained from 3 different consultations, documented in the participant´s medical records. The severity of hypertension was defined as SBP of 140-159mmHg or a DBP 90-99mmHg for stage 1 and SBP ≥160mmHg or diastolic blood pressure (DBP) ≥100mmHg for stage 2 JNC 7 [14]. The antihypertensive drug prescription considered was the most recent prescription. The control of hypertension was defined according to the Eighth Joint National Committee (JNC8) as BP <140/90mmHg in hypertensive patients <60 years old, diabetic patient or patients with renal failure and BP <150/90mmHg in hypertensive patients ≥60 years old who are not diabetic and without renal failure [12]. Monotherapy was defined as treatment containing a single active ingredient. Combination therapy was defined as treatment containing either two active ingredients (bitherapy), three active ingredients (tritherapy) or four active ingredients (quadritherapy). A fixed-drug combination was defined as a drug containing two or more active ingredients in a single tablet. Participants who smoked at least one cigarette per day at the time of the study were classified as current smokers, and those who had smoked for at least 3 years in the past but had stopped by the time of the study were classified as ex-smokers. Prevalent diabetes was based on ongoing antidiabetic drugs or a documented history of diabetes.

**Data analysis:** categorical variables are expressed as frequencies and percentages while quantitative variables are expressed as mean and standard deviation for normally distributed variables and median and 25^th^-75^th^ percentiles for skewed variables. The chi-square and Fischer exact test were used to compare categorical variables as appropriate in the univariate analysis. Variables that were statistically significant in the univariate model were used for analysis in the multivariate analysis. Logistic regressions adjusted for age, gender and center were used to determine the significant predictors of prescription of antihypertensive medications and BP control. For the prescription of antihypertensive medications, the predictor variables were; the age, duration of hypertension, duration of practice, the severity of hypertension, average monthly income, prescriber, presence of comorbidities and the dependent variable was the different treatment regimen. When assessing factors that influence BP control, predictor variables were; age, gender, marital status, occupation, physical activity, smoking, BMI and treatment regimen while the dependent variable was the level of BP control. All statistical analyses were performed using SPSS version 23 IBM and p-value ≤0.05 was taken to indicate significance.

**Ethical consideration:** ethical clearance was obtained from the Institutional Review Board of the Faculty of Health Sciences, the University of Buea and the study conformed to the principles outlined in the Declaration of Helsinki. Written informed consent was obtained from each eligible participant before inclusion.

## Results

**Figure 1:**
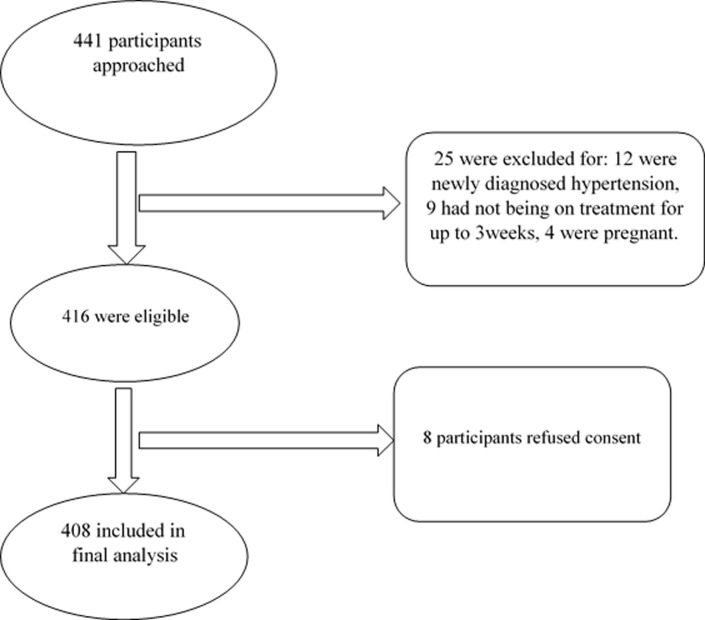
shows the flow process for the recruitment of participants in the current study

**General characteristic of the study population:** a total of 408 participants were included in the study (Figure 1). The mean age of participants was 61.1 ± 12.2 years (minimum-maximum 21-98 years); 272 (63.7%) were female, 240 (59.4%) were married, 207 (51.1%) were unemployed and 153 (38.5%) had primary education and 41 (10.3%) had no formal education (Table 1). The median diagnosed duration of hypertension was 6 years and the median duration of current treatment was 22.0 weeks. In all, 76.2% of the participants had associated comorbidity, while 352 (87.3%) were receiving treatment from a doctor (Table 2).

**Table 1:** socio-demographic characteristics of the study population

Characteristics	Male (n=136)	Female (n=272)	p-value
Mean Age (±SD), years	61.1 (±11.6)	61.1 (±12.5)	0.984
**Age groups, n (%)**			0.495
20–39	7 (5.1)	14 (5.1)	
40–59	45 (33.1)	105 (38.6)	
60–79	80 (58.8)	140 (51.5)	
80–100	4 (2.9)	13 (4.8)	
**Marital status n (%)**			**<0.001**
Single	5 (3.7)	35 (12.9)	
Married	115 (84.6)	125 (46.0)	
Divorced	1 (0.7)	6 (2.2)	
Widow(er)	14 (10.3)	103 (37.9)	
**Level of education n (%)**			**<0.001**
No formal education	6 (4.4)	45 (16.5)	
Primary	41 (30.1)	114 (41.9)	
Secondary	49 (36.0)	64 (23.5)	
Tertiary	40 (29.4)	49 (18.0)	
**Occupation n (%)**			**<0.001**
Employed	59 (44.0)	75 (27.7)	
Unemployed	40 (29.9)	167 (61.6)	
Retired	35 (26.1)	27 (10.0)	
Student	0	2 (0.7)	

**Table 2:** clinical characteristics of the study population

Characteristics	Male	Female	p-value
Median duration of hypertension (25^th^-75^th^ percentiles), years	5 (3-10)	6.5 (3-12)	0.397
^*^Mean monthly income ($)	313.6	214.7	0.071
Median duration of current treatment (25^th^-75^th^ percentiles), weeks	20 (7.3-52)	24 (8.1-54.5)	0.266
Median duration of follow-up (25^th^-75^th^ percentile), months	12 (5-24)	12 (6-36)	0.527
Mean SBP (±SD), mmHg	143.5 (±24.2)	141.4 (±21.4)	0.374
Mean DBP(±SD), mmHg	85.4 (±13.3)	86.7 (±12.5)	0.337
Associated comorbidities /risk factors			
Smoking n (%)	15 (11.0)	1 (0.4)	**<0.01**
Body Mass Index n (%)			**0.001**
Normal	37 (32.7)	43 (18.7)	
Overweight	45 (39.8)	79 (34.3)	
Obese	31 (27.4)	108 (47.0)	
Diabetes mellitus n (%)	85 (63.0)	157 (57.9)	0.387
History of chronic kidney disease n (%)	116 (85.3)	248 (91.2)	0.102
History Heart failure n (%)	125 (92.6)	245 (90.4)	0.586
History Ischemic Heart disease n (%)	125 (93.3)	257 (95.2)	0.575
History of Stroke n (%)	117 (86)	245 (90.4)	0.246

* conversion unit from CFA to dollars was 581

**Figure 2:**
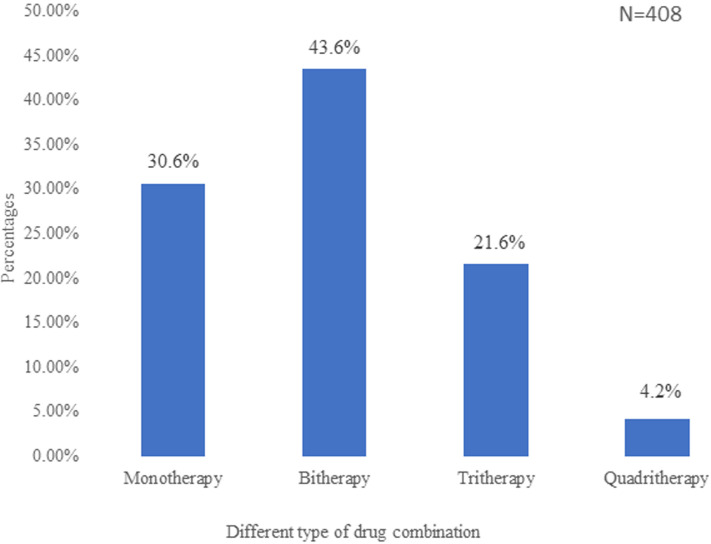
distribution of the different drug combinations

**Prescription patterns of antihypertensive drugs:** seventy-two percent of participants were prescribed the proprietary form of drugs, 16% prescribed the generic form and 12% were prescribed both the generic and proprietary forms. The median number of drugs received by the participants was one. The average monthly cost of antihypertensive medication was 17.7 ± 13.7 dollars. Two hundred and eighty-two (71.9%) of participants paid for their drugs from out of pocket money while only 4 (1.0%) benefitted from insurance coverage. In all, 93.1% of patients were prescribed a once-daily drug regimen. Of the 408 prescriptions, 44% were bitherapy, 31% monotherapy and 26% contained three or more drugs (Figure 2). Among the 283 participants on combination therapy, 121(42.8%) were on fixed combinations.

**Figure 3:**
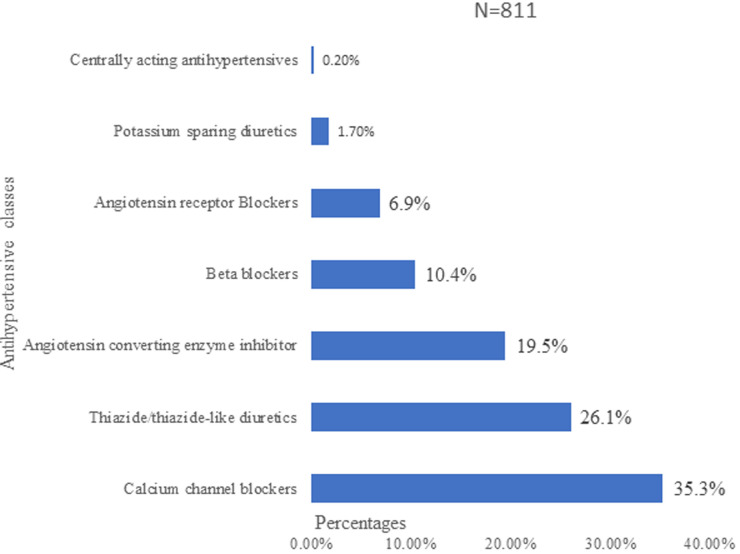
distribution of different classes of anti-hypertensive drugs prescribed

Calcium channel blockers (CCB) were the most prescribed antihypertensive in 35.1% of the participants either as single or combination therapy with the most frequent molecule been amlodipine (30.5%). Thiazide/thiazide-like diuretic (TD/TLD) in 26.1% was the second most prescribed therapeutic class, with the most frequently used molecule being hydrochlorothiazide (HCT,14.6%); while angiotensin-converting enzyme inhibitors (ACEI) in 19.5% was the third leading class, with the most frequently used molecule been perindopril (7.0%); (Figure 3). Among participants on combination therapy, ACEI+TD/TLD (19.4%), CCB+TD/TLD (18.7%) and ACEI+CCB+TD/TLD (10.6%) were the most frequently used combinations. The severity of hypertension, duration of hypertension and the presence of comorbidity were the independent determinants of prescription of antihypertensive drugs (Table 3).

**Table 3:** multivariable logistic regressions adjusted for age, sex and center

Variables	Bitherapy OR (95% CI)	Tritherapy OR (95% CI)	Quaditherapy OR (95% CI)
Age of patient < 60 years	1.8 (0.9-4.0)	2.2 (0.9-5.4)	4.1 (0.8-22)
Age of doctor < 39 years	1.4 (0.8-2.4)	1.6 (0.8-2.9)	2.7 (0.9-8.1)
Duration of practice < 12 years	0.9 (0.5-1.7)	1.2 (0.6-2.4)	2 (0.6-6.2)
Duration of hypertension < 6 years	0.6 (0.5-1.2)	**0.4 (0.2-0.7)**	0.4 (0.1-1.1)
Severity of hypertension			
Normal	0.9 (0.4-1.9)	1.8 (0.6-5.1)	1.5 (0.3-9.2)
Mild	1.4 (0.9-2.4)	**4.5 (2.2-9.2)**	2.2 (0.7-7.4)
Prescribing doctor			
General practitioner	0.8 (0.4-1.4)	0.5 (0.2-1.1)	0
Patient's monthly income < 173$	1.1 (0.4-2.7)	0.2 (0.6-5.8)	0.6 (0.1-3.4)
History of diabetes	1.3 (0.8-2.1)	**1.8 (1.1-3.4)**	1.1 (0.4-3.1)
History of stroke	1.1 (0.5-2.4)	0.6 (0.3-1.5)	1.8 (0.2-15)
History of chronic kidney disease	0.5 (0.2-1.2)	0.7 (0.3-2.1)	0.3 (0.1-1.4)
History of heart failure	1.3 (0.5-3.4)	0.5 (0.2-1.3)	**0.2 (0-0.5)**
History of ischaemic Heart Disease	0.3 (0.1-1.5)	0.3 (0.05-1.5)	**0.02 (0.0-0.1)**

Reference is monotherapy

**Blood pressure control:** in all, 211 (51.7%) participants had a controlled level of BP; with no gender difference in proportions of controlled hypertension (50% in men Vs 52.6% in women p=0.70). By treatment regimens, the proportion of controlled hypertension was 60% for monotherapy, 50.3% for bitherapy, 48.3% for tritherapy and 23.5% for quadritherapy (p=0.024). The control rate was higher in participants aged ≥60 years than in those aged <60 years (59.9% vs 40.4%, p <0.01), and varied by BMI status (p=0.046). In age, sex and center adjusted logistic regression, BMI <25 (OR 2.3;1.2-4.3), been on monotherapy (OR 5.2;1.6-17.3) and Bitherapy (3.4;1.1-11.3) were significant predictors of BP control.

## Discussion

Our study revealed that, calcium channel blockers (CCB) were the most commonly prescribed antihypertensive in a third of participants, followed by thiazide/thiazide-like diuretic (TD/TLD) and angiotensin-converting enzyme inhibitor (ACEI). About two-thirds of participants were on combination therapy, with the severity of hypertension and duration of hypertension being the predictor of prescription. About half of treated patients were at target BP control level with the age of patients, BMI and treatment regimen being important correlates of BP control. The patterns of prescription in our study were similar to those found by Menanga *et al*. in a study in urban Cameroon in 2016 [25]. They are also in line with JNC on Prevention, Detection, Evaluation, and Treatment of high blood pressure guidelines which recommend the use CCB and Thiazide/thiazide- like diuretics as first-line drugs in the management of hypertension among blacks [12]. However, a dissimilarity was found in the prescription of antihypertensive in a study done by Adejumo *et al*. and Busari *et al*. in Nigeria who found that diuretics were the commonly prescribed antihypertensive medications [26, 27]. This difference could be due to the availability and accessibility of CCB in our setting and the fact that the majority of our patients were elderly. Although diuretics which are relatively cheaper were traditionally the mainstay of hypertension management in this age group, other classes of antihypertensives such as CCB, ACEI and sympatholytic have proven to be beneficial in this age group either in single or combination therapy with little side effects [28].

The percentage of participants on combination therapy (70%) was similar to that reported by Adedapo *et al*. in Nigeria (79%) [29] but higher than the 50% reported by Menanga *et al*. in Ethiopia (50%). A higher proportion of participants in our study had comorbidities just like participants in Adedapo *et al*. study (77% and 88% respectively) whereas only 28% of participants in Menanga *et al*. study had associated comorbidities [25]. The JNC on Prevention, Detection, Evaluation, and Treatment of high BP guidelines advise that: more than 50% of patients with hypertension will require more than one drug to control their BP [30]. Studies have shown that in the presence of comorbidities coupled with other factors such as the severity of hypertension and presence of end-organ damage, the use of multiple antihypertensive drugs is likely beneficial particularly when used at low doses to reduce the side effect that may occur with higher doses of a single drug [31]. The most common drug combination used were ACEI+TD/TLD, CCB+TD/TLD and ACEI+CCB+TD/TLD which is similar to that of Menanga *et al*. in Cameroon and Adejumo *et al*. in Nigeria and are both in accordance with the JNC 8 guidelines [12, 25, 26]. Even though the combination of 2 or more antihypertensives at fixed-dose in a single tablet is recommended and has a beneficial effect on improving adherence to medication: 57.2% of our participants were on free drug combination. This high value could be accounted for by the very few available fixed-dose combinations as tritherapy or quadritherapy.

In our setting, the only available fixed drug combination as tritherapy was either ACEI+CCB+TD/TLD or ARB+CCB+TD/TLD which was relatively expensive when compared to a similar combination containing separate pills. Controlling hypertension is very challenging in SSA with only 7% of people with hypertension having their BP below targeted values [32]. In a previous nationwide study, we also reported a lower level of hypertension control among Cameroonian hypertensive with only24.6% having their BP at target levels [33]. The control rate of hypertension among our participants was high (52%), the reason being that a higher threshold of BP control was used in this study [13]. Given that recent JNC 8 guidelines recommend a greater threshold for control BP in patients >60 years (SBP/DBP <150/90mmHg), much of our participants were beyond this age group. Also, the higher threshold for BP controlled among diabetic patients (SBP/DBP <140/90mmHg) compared to the previous cut off SBP/DBP <130/80mmHg could also account for this high control rates. Furthermore, a greater proportion of participants in our study already had associated comorbidities/complications related to hypertension. These likely increased their adherence to regular follow up visits, taking their treatment and they could also benefit from health talks and counseling given by health personnel. These findings are much higher than those by Alba-Leonel *et al*. (37.4%) [34]. This could be accounted for by the different definitions of BP control used in these studies which rather defined BP control as <140/80mmHg irrespective of the patient´s age or BP <130/80mmHg in diabetic patients.

However, the control rate of participants was much similar to that of Adejumo *et al*. [26] in Nigeria (53.6%) who used similar BP cut off to that in our study and 50.3% of Riley *et al*. in Ethiopia [24]. Previous studies in both developing and developed countries have shown the BP control rate to range from 15.5%-72% [34-37]. The higher BP control rate among participants in developed countries could be explained by better medical care, better education, better awareness of patients to hypertension and treatment in these countries [38]. Up to 60% of participants on monotherapy had their BP controlled. Controlled BP can be achieved by monotherapy in patients with mild hypertension compared to those with moderate to severe hypertension which will require combination therapy. More so, the pill burden with a single drug is far less demanding and it is associated with better compliance, fewer side effects or drug interaction and lower costs. This finding was similar to that of Adejumo *et al*. (64%) but however much higher than that obtained by Busari *et al*. who (34%) [26, 27]. The smaller proportion of participants on monotherapy could account for this difference in the study done by Busari *et al*. Most studies have shown that patients will require 2 or more antihypertensive to achieve BP control [39-41].

**Study limitations:** blood pressure control was assessed using office BP reading which may tend to underestimate the BP control rate. Also, this study was a hospital-based study done in a secondary care setting, so may not reflect the situation in the primary health care setting. Adherence to antihypertensive medication wasn´t assessed in this study.

## Conclusion

Contemporary antihypertensive prescription patterns in our study were dominated by CCB, diuretics and ACEI with 7 of every 10 patients receiving at least 2 drugs. Half of the patients had their BP controlled which is still below optimal but far better than previous reports. The need to intensify all efforts geared towards BP control to reduce the morbidity, mortality and cost burden associated with hypertension cannot be overemphasized. As such, providing clinicians with simple, clear and concise local hypertension guidelines with peculiarities on first-line medications and combination pharmacotherapy is critical. Also, health policymakers need to improve the availability and affordability of required medications so as to design more effective interventions that improve hypertension control and management.

### What is known about this topic

- Uncontrolled hypertension is a major health issue in sub-Saharan Africa with only 7% of hypertensive patients on treatment have their BP under control;
- Prescription of antihypertensive drugs prevents adverse complications of hypertension such as stroke, myocardial infarction and heart failure;
- Several international guidelines recommend a combination of 2 or more drugs from different classes in order to achieve optimal BP control with little or no place for monotherapy.

### What this study adds

- There is the absence of local guidelines to treat hypertension in several African countries and furthermore, little was known on the prescription of pharmacotherapy and its effectiveness on BP control among hypertensive blacks leaving in SSA; in our study, CCBs were the most prescribed single antihypertensive drugs;
- Monotherapy was effective in controlling BP in over 60% of our patients with uncomplicated and non-severe hypertension. Hence there is still a place for a single-drug regimen in controlling uncomplicated hypertension; clinicians in our setting tend to prescribe more combination therapy as recommended by most contemporary guidelines, especially in the presence of comorbidities;
- Although 70% of patients were treated with a combination of 2 or more drugs, BP control of hypertension could be achieved in only 50% of our participants; this study emphasizes the need for early detection, treatment and control of hypertension using combination therapy. Providing clinicians with local guidelines to support their practice in a bit to help reduce the burden of uncontrolled hypertension.
